# A correlative approach for combining microCT, light and transmission electron microscopy in a single 3D scenario

**DOI:** 10.1186/1742-9994-10-44

**Published:** 2013-08-03

**Authors:** Stephan Handschuh, Natalie Baeumler, Thomas Schwaha, Bernhard Ruthensteiner

**Affiliations:** 1 VetImaging, VetCore Facility for Research, University of Veterinary Medicine, Veterinärplatz 1, 1210, Vienna, Austria; 2 Department of Theoretical Biology, University of Vienna, Althanstraße 14, 1090, Vienna, Austria; 3 Konrad Lorenz Institute for Evolution and Cognition Research, Adolf Lorenz Gasse 2, 3422, Altenberg, Austria; 4 Zoologische Staatssammlung München, Münchhausenstr 21, 81247, Munich, Germany; 5 Department of Integrative Zoology, University of Vienna, Althanstraße 14, 1090, Vienna, Austria

## Abstract

**Background:**

In biomedical research, a huge variety of different techniques is currently available for the structural examination of small specimens, including conventional light microscopy (LM), transmission electron microscopy (TEM), confocal laser scanning microscopy (CLSM), microscopic X-ray computed tomography (microCT), and many others. Since every imaging method is physically limited by certain parameters, a correlative use of complementary methods often yields a significant broader range of information. Here we demonstrate the advantages of the correlative use of microCT, light microscopy, and transmission electron microscopy for the analysis of small biological samples.

**Results:**

We used a small juvenile bivalve mollusc (*Mytilus galloprovincialis*, approximately 0.8 mm length) to demonstrate the workflow of a correlative examination by microCT, LM serial section analysis, and TEM-re-sectioning. Initially these three datasets were analyzed separately, and subsequently they were fused in one 3D scene. This workflow is very straightforward. The specimen was processed as usual for transmission electron microscopy including post-fixation in osmium tetroxide and embedding in epoxy resin. Subsequently it was imaged with microCT. Post-fixation in osmium tetroxide yielded sufficient X-ray contrast for microCT imaging, since the X-ray absorption of epoxy resin is low. Thereafter, the same specimen was serially sectioned for LM investigation. The serial section images were aligned and specific organ systems were reconstructed based on manual segmentation and surface rendering. According to the region of interest (ROI), specific LM sections were detached from the slides, re-mounted on resin blocks and re-sectioned (ultrathin) for TEM. For analysis, image data from the three different modalities was co-registered into a single 3D scene using the software AMIRA®. We were able to register both the LM section series volume and TEM slices neatly to the microCT dataset, with small geometric deviations occurring only in the peripheral areas of the specimen. Based on co-registered datasets the excretory organs, which were chosen as ROI for this study, could be investigated regarding both their ultrastructure as well as their position in the organism and their spatial relationship to adjacent tissues. We found structures typical for mollusc excretory systems, including ultrafiltration sites at the pericardial wall, and ducts leading from the pericardium towards the kidneys, which exhibit a typical basal infolding system.

**Conclusions:**

The presented approach allows a comprehensive analysis and presentation of small objects regarding both the overall organization as well as cellular and subcellular details. Although our protocol involves a variety of different equipment and procedures, we maintain that it offers savings in both effort and cost. Co-registration of datasets from different imaging modalities can be accomplished with high-end desktop computers and offers new opportunities for understanding and communicating structural relationships within organisms and tissues. In general, the correlative use of different microscopic imaging techniques will continue to become more widespread in morphological and structural research in zoology. Classical TEM serial section investigations are extremely time consuming, and modern methods for 3D analysis of ultrastructure such as SBF-SEM and FIB-SEM are limited to very small volumes for examination. Thus the re-sectioning of LM sections is suitable for speeding up TEM examination substantially, while microCT could become a key-method for complementing ultrastructural examinations.

## Background

In biomedical research, a huge variety of different techniques is currently available for the structural examination of small specimens. In small invertebrate animals, cellular details and overall organization are often examined by conventional brightfield light microscopy (LM), which can also be extended to a three-dimensional analysis by the examination of section series (e.g. [1-3]). While conventional LM is limited in resolution to approximately 0.2 μm, transmission electron microscopy (TEM) provides much higher resolutions allowing the investigation of subcellular details (e.g. [4]). The use of specific markers and fluorescent dyes in combination with conventional fluorescence microscopy or confocal laser scanning microscopy (CLSM) allows for assessment of specific tissues or cellular components (e.g. [5]), and microscopic X-ray computed tomography (microCT) allows imaging of the X-ray dense structures of entire specimens (e.g. [6]).

It is becoming increasingly popular to combine some of these and other imaging methods for specific research questions. This combined approach for examination of a single specimen is usually termed correlative microscopy (e.g. [7,8]). A variety of combinations such as CLSM with TEM (e.g. [9,10]), LM with scanning electron microscopy (SEM) and TEM (e.g. [11]), microCT with LM (e.g. [12]), or microCT with CLSM [13] have been applied so far. Recently, an approach was presented that correlates LM and TEM images to single microCT sections [14]. The most common combination is LM with TEM, which has received its own acronym, correlative light and electron microscopy (CLEM) (e.g. [8,15]). CLEM includes all attempts of re-sectioning LM sections for TEM investigations that use LM sections for tracking down ROIs for further investigation. The merits of all these methodological combinations come from a broadening of the range of information and a reduction of the total workload.

Small specimens routinely processed for TEM are ideally suited for correlative approaches. Such specimens are typically post-fixed with osmium tetroxide, which increases electron density of soft tissues and thus contrast for both TEM examination and X-ray microCT scanning [14,16]. Hence, specimens prepared for TEM can be examined directly by microCT without further treatment [17,18]. Specimens treated with osmium tetroxide postfixation are also routinely used for LM serial section analysis (e.g. [19]) and re-sectioning of LM sections for TEM is an established technique (e.g. [20]). Accordingly, no specific preparatory processes are required for a combined examination by microCT, LM serial sections and TEM of the same specimen. Such a threefold approach seems highly promising because it provides information of different kinds and at different spatial scales, substantially increasing the information gained on the organization of the specimen in general.

Most recent studies with a correlative approach apply 3D analysis and visualization. The datasets of the individual methods are usually treated separately [11,21]. However, datasets can be connected to each other directly in a single scene. State-of-the-art commercial 3D software enables a co-registration of datasets, i.e. aligning size and position in one 3D coordinate system for simultaneous display. For example, Lucas et al. [22] show perfectly co-registered CLSM and TEM datasets. In this example co-registration procedures are relatively simple, because the axes of the datasets are orthogonal. As we will illustrate, more complex processes for co-registration, including the rotation of datasets, work well too.

To illustrate the merits of co-registration of microCT, LM, and TEM data we use a juvenile bivalve mollusc. Our analysis includes the microCT volume dataset, the volume dataset and segmentation-based surface models of the serial LM section images, and selected TEM section images. The muscular, vascular, excretory, and digestive systems were chosen for segmentation and surface rendering. The excretory organs were selected for the TEM investigation because their size relative to the entire specimen is typical for fine structural investigations, they bear complex ultrastructural details, and their detailed organization contributes information to ongoing studies on the nephrogenesis of molluscs [23,24].

## Methods

### Overview of the methods applied

**Figure 1:**
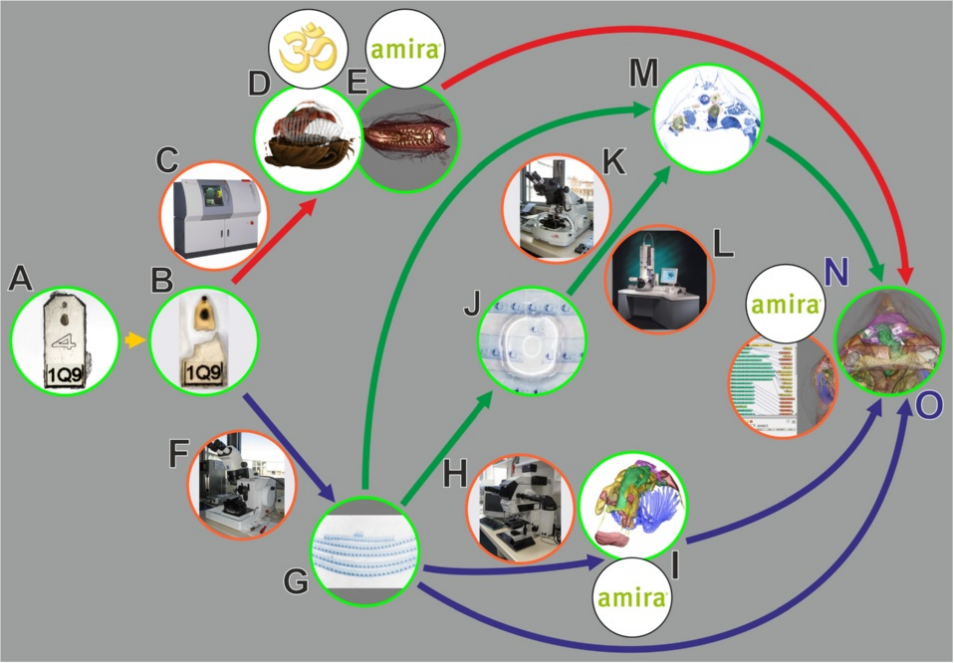
**General workflow for combining microCT, LM and TEM.** Red arrows, volume data from microCT; blue arrows, LM section data; green arrows, TEM section data; green encircled, material and (interim) results; orange encircled, microscopic equipment; black encircled, software systems. **A**. EM fixed specimen embedded in epoxy resin block, anterior one used in study. **B**. Block trimmed and anterior portion detached, after microCT scan. **C**. MicroCT. **D**. Volume rendering with Drishti. **E**. Volume rendering with AMIRA®. **F**. Microtome for LM sectioning. **G**. LM section series. **H**. Light microscope. **I**. Surface rendering with AMIRA®. **J**. Remounting of LM sections for ultrathin sectioning. **K**. Ultramicrotome. **L**. TEM. **M**. 2D alignment for figure plates. **N**. 3D registration with AMIRA®. **O**. Combined 3D visualization of all datasets.

The specimen was fixed and embedded using standard procedures for transmission electron microscopy (TEM) investigation. Subsequently it was scanned by microCT. This was followed by sectioning for light microscopical (LM) investigation. Based on serial LM images and image segmentation, a 3D surface model of organ systems was generated. This permitted precisely tracking down ROIs and thus individual LM sections for fine structural investigation. Selected sections were re-sectioned for TEM. The datasets gained from microCT (volume dataset), LM (volume dataset, surface model) and TEM (individual sections) were co-registered and combined to a single visualization using AMIRA® (version 5.3.3, Visage Imaging, Berlin, Germany) (Figure 1).

### Sample treatment and laboratory procedures

#### Collection, fixation, embedding

The juvenile specimen of *Mytilus galloprovincialis* Lamarck, 1819 was collected on the shore near the Observatoire Océanologique de Banyuls-sur-Mer, France, in spring 2009. It was anesthetized with magnesium chloride and fixed for several days in 4% glutaraldehyde in 0.2 mol l^-1^ cacodylate buffer, followed by 2 h postfixation in 1% osmium tetroxide in 0.2 mol l^-1^ cacodylate buffer. After decalcification in 1% ascorbic acid, the specimen was dehydrated in an ascending acetone series (further details given in [25]) and embedded in an epoxy resin (Agar Low Viscosity Resin Kit, Agar Scientific, Stansted, England).

#### MicroCT

**Figure 2:**
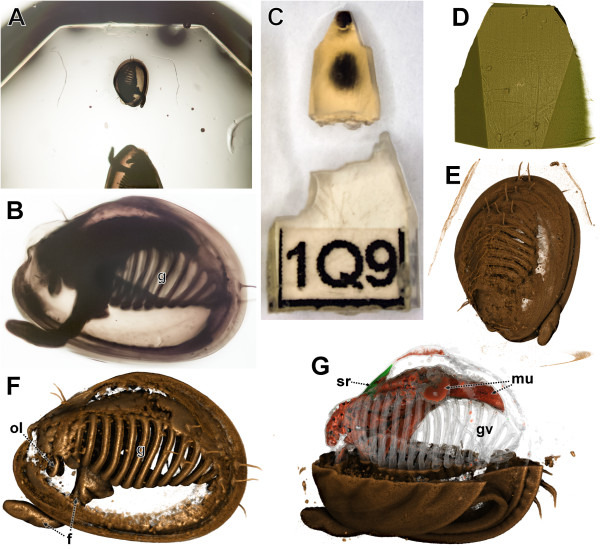
**Specimen embedded in block and volume rendering. A**. Anterior part of block, anterior (upwards) specimen used for study. **B**. Photograph of specimen in block. **C**. Block trimmed and anterior portion detached, after microCT scan. **D**–**G**. Volume rendering with Drishti software. **D**. Transfer functions set to view embedding resin, facing bottom of block. **E**, **F**. Different transfer function settings. **G**. Several independent transfer function setting optimized for specific specimen structures with clipping part of the transfer function results. f, foot; g, gill; gv, gill vessels; mu, muscle; ol, oral lappets; sr, shell resilium.

Prior to microCT scanning the resin block was trimmed to a shape as required for sectioning. Subsequently a part of the block was removed to facilitate mounting in the microCT scanner (Figure 2C,D). Scanning was performed with a Nanotom (GE Sensing & Inspection Technologies GmbH, Wunstorf, Germany) at 50 kV for 4.3 hours. 1,440 projection images yielded a volume dataset with the dimensions of 650×638×838 with 1.2 μm (isotropic) voxel size. By the CT scanning process the resin of the block was considerably darkened (Figure 2C). However, this had no the effect on the following TEM sectioning and examination procedures.

#### Sectioning

**Figure 3:**
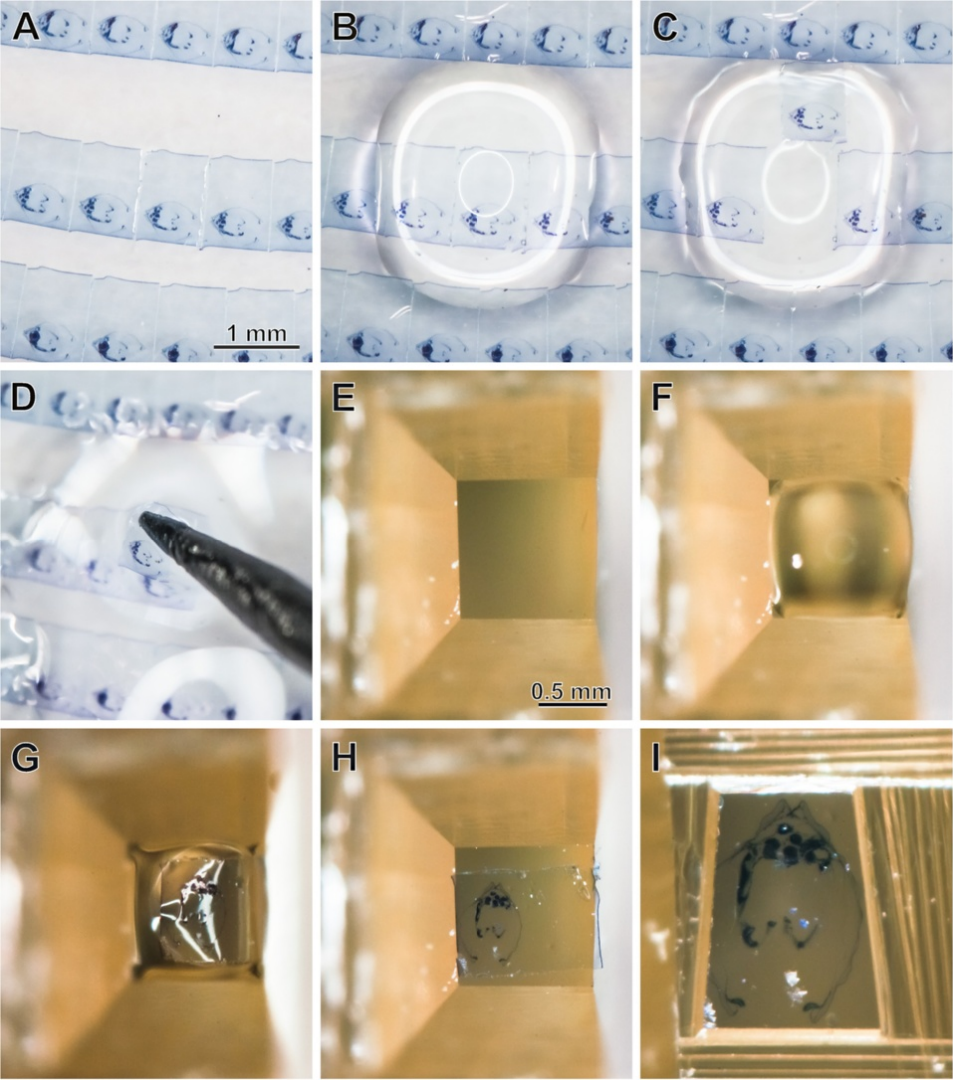
**Re-sectioning of LM sections for TEM. A**. Excerpt of the LM section series on slide. **B**. Drop of distilled water is placed atop the section to be lifted. **C**. Section detached. **D**. Section is picked up with the tip of a needle. **E**. Block that has been perfectly smoothened by sectioning. **F**. Drop of distilled water placed atop the block. **G**. Section placed in drop. **H**. Section dried on block surface. **I**. Block trimmed for TEM re-sectioning.

The specimen was initially sectioned for LM (“semithin”) at a thickness of 1.5 μm using a Histo Jumbo diamond knife (Diatome AG, Biel, Switzerland) and with ribbon formation of sections (see [25] for protocol). The section ribbons were applied to conventional (un-pretreated) microscope slides that were cleaned as described by [25]. Since some sections later had to be detached for TEM investigation, the series was left uncovered with no mounting medium and no coverslip applied. Re-sectioning of LM sections for TEM (Figure 3) followed the method described by Campbell & Hermans [26]. An empty resin block was trimmed so that it had a cutting surface as large as the LM section to be re-mounted. The empty block was sectioned on an ultramicrotome with a diamond knife to obtain a smooth surface (Figure 3E). The holder with the block was then removed from the microtome and placed with the cutting surface facing up and a drop of distilled water was placed on it (Figure 3F). Subsequently the LM sections selected for re-sectioning were removed from the microscope slide by placing a drop of distilled water at the edge of the section (Figure 3B). The section then was removed from the slide by gently detaching it from the side with the tip of a fine needle and simultaneously dragging the water underneath the section (Figure 3C). This resulted in the section floating on the surface of the drop. From here sections were picked up with tip of a needle (Figure 3D) and transferred to the drop on top of the cutting surface of the block (Figure 3G). Excess water was removed with the help of filter paper to prevent wrinkles in the sections. Thereafter the block was placed in an oven at 40°C for at least 30 minutes to increase adhesion of the section to the block (Figure 3H). Prior to TEM sectioning the block was trimmed until the cutting face was smaller than the LM section before re-mounting (Figure 3I); every edge of the LM section was cropped.

For ultrathin sectioning only slight re-adjustment of the cutting angle was required, since settings were retained from smooth sectioning of the empty block before mounting the LM section. Ultrathin sectioning was performed with a diamond knife. Each LM section yielded up to 13 TEM sections of 50–70 nm thickness and reasonable quality.

The TEM sections were mounted on formvar coated slot grids. They were stained with uranyl acetate and lead citrate solutions with the help of a grid staining matrix system (Ted Pella, Inc., Redding, CA, USA), and examined using an FEI Morgagni 268 TEM (FEI Company, Eindhoven, Netherlands) equipped with an Olympus MegaView III digital camera (Olympus Soft Imaging Solutions GmbH, Münster, Germany) at 80kV and a resolution of 1.3 megapixels. Individual exposures were automatically stitched; every image used for figures of this study consists of at least four exposures.

### Image processing and 3D visualization

#### MicroCT dataset

The microCT dataset was visualized by volume rendering with AMIRA® (see also below) and DRISHTI 2.x [27] (Figure 2D–G) software. In DRISHTI we applied transfer functions in the *2D histogram*. Individual color and transparency settings for multiple transfer functions permitted discerning tissues with different density attributes. With the help of *ClipPlanes* individual parts were selectively hidden (Figure 2G, Additional file 1).

#### LM image acquisition, segmentation and surface rendering

Images of the 585 sections of the LM series were taken with a Spot Insight camera (Diagnostic instruments Inc., Sterling Heights, USA) mounted on a Leica (Leica Microsystems, Wetzlar, Germany) DMB-RBE microscope at a resolution of 1,600×1,200 pixels. The images were set to 8-bit grayscale and enhanced with Photoshop CS5 (Adobe Systems Incorporated, 345 Park Avenue, San Jose, CA 95110–2704). Slice alignment, segmentation and generation of surface meshes were performed with the software AMIRA®, mostly following the procedures outlined by Ruthensteiner [25]. Since no cropping was required, the dimensions of the final LM section image stack remained at 1,200×1,600×585 (voxel size: 0.413×0.413×1.5 μm). The renopericardial system, the nervous system, the digestive system, muscles, and the gill vessels were segmented.

#### Affine co-registration of image stacks

**Figure 4:**
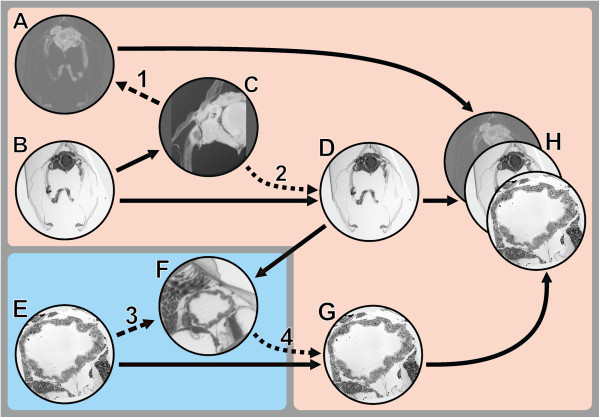
**Workflow of TEM section 3D registration. A**. MicroCT stack. **B**. LM image stack. **C**. LM image stack inverted and Gaussian filtered to enhance similarity with the microCT stack. **D**. Untreated LM stack with co-registration parameters adopted from the previously co-registered LM stack that was inverted and filtered. **E**. TEM section. **F**. Template of LM section that was re-sectioned for TEM. **G**. TEM section with 3D co-registration parameters. **H**. Final 3D scenario with co-registered data of microCT, LM and TEM. Processes: 1, co-registration of the modified LM image stack with the microCT stack; 2, adoption of the co-registration parameters to the untreated LM stack; 3, 2D registration of TEM images in LM image templates with the help of Photoshop. 4, adoption of the of the 3D co-registration parameters to the 2D registered TEM image. Blue background: 2D environment; peach colored background: 3D environment.

For co-registration of image stack data from two different sources (microCT and LM serial section images, Figure 4A,B) both stacks were loaded into the AMIRA® *Pool* and saved in amira mesh (AM) format. The microCT stack was used as reference dataset, as it is free from both geometric distortions and misalignments. The LM stack (the same as previously processed for segmentation – see above) was then inverted using the *Arithmetic* module (*expr:* A*–1+255) and filtered using *Gauss-Smoothing* (3D, *Kernel*: 3/3/3) (Figure 4C). These steps enhanced similarity to the CT dataset and thereby facilitated co-registration based on correlation metrics for use with the *AffineRegistration* module. Subsequently the LM image stack was coarsely aligned with the reference (microCT) stack by hand (while displaying both stacks with a *Voltex* module) using the *Transform Editor*. This was followed by fine co-registration, which was performed automatically with the *AffineRegistration* module (Additional file 2). This module was connected to the LM stack and the *Reference* port was connected to the microCT stack. The only parameter changed from the default settings was *Correlation* (at *metric*). Thus, the registration process was rigid and included subsequent steps of rotation and translation. After registration, the transformed section image stack was saved. The same transformation parameters were subsequently applied (copy/paste in the *Transform Editor* dialog) to the original (non-inverted, unfiltered) LM stack (Figure 4D) and to the segmentation stack. Accordingly, both LM stacks, the segmentation stack as well as the surfaces resulting from the segmentation dataset became co-registered with the microCT stack in the 3D scene.

#### Registration of TEM images

**Figure 5:**
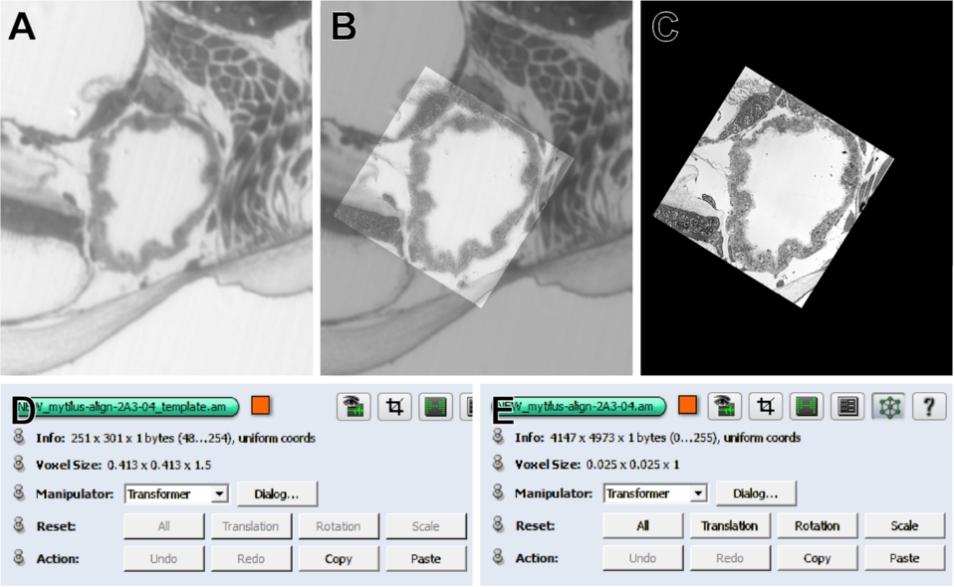
**TEM section 3D registration. A**. Template from an LM section that was used for TEM re-sectioning. **B**. TEM image co-registered to the LM template in Photoshop. TEM image layer set to 50% opacity. **C**. Registered TEM image with (nearly) black background as re-imported into AMIRA®. **D**. Voxel dimensions (0.413×0.413 μm in X and Y axes) of the cropped LM template before resampling to TEM resolution. **E**. Voxel dimensions (0.025×0.025 μm) of the cropped LM template after resampling to TEM resolution (same as in 3D registered TEM image).

Prior to the registration of a TEM image into the 3D *network*, we created templates from the LM sections that were actually used for TEM re-sectioning (Figure 4F). To achieve this, the selected LM section image was isolated from the already registered LM stack with the *Crop Editor* and saved as separate AM file in AMIRA®. For each TEM image to be registered, the LM section was cropped to the specific region of interest (Figure 5A) again with the *Crop Editor*. Subsequently the resolution of the dataset (crop of a single slice) was strongly increased (*Compute, Resample*) to reach the voxel (pixel) size of the respective TEM image (calculated from TEM scale bar) (Figure 5D and 5E). After resampling of the template, the transformation details had to be restored from the original cropped image using the copy/paste function of the *Transform Editor*, and the resampled and transformed template was saved in AM format. The template was then exported in 2D TIFF format and loaded into Photoshop CS5. Thereafter the TEM image was loaded into the same Photoshop document into a new layer and aligned to the LM template using the *Auto-Align-Layers* function. To check the quality of alignment, the TEM image layer was inspected with 50% opacity (Figure 5B). Distortion of TEM images relative to the LM images was mostly negligible and no elastic registration appeared necessary. In a few cases the size of TEM images had to be slightly rescaled (isotropically) to match the LM image exactly. A black background layer was inserted below the TEM image layer, and the 8-bit histogram of the TEM image was modified to 10–255 using a *Levels layer* to provide that all areas of the TEM image remain visible at visualization in AMIRA® (see below). Eventually the registered TEM image was combined with the fully black background and saved as TIFF file (Figure 5C; note that it is crucial that the canvas size in the Photoshop document remains unchanged during this procedure). This image was then re-imported into AMIRA®. The position coordinates within the scenery were restored from the resampled template with the *Crop Editor*, and transformation coordinates were reused from the resampled template again using the copy/paste function of the *Transform Editor*. Finally the TEM image was saved in Amira mesh (AM) format.

#### Visualization of the 3D scene in AMIRA®

**Figure 6:**
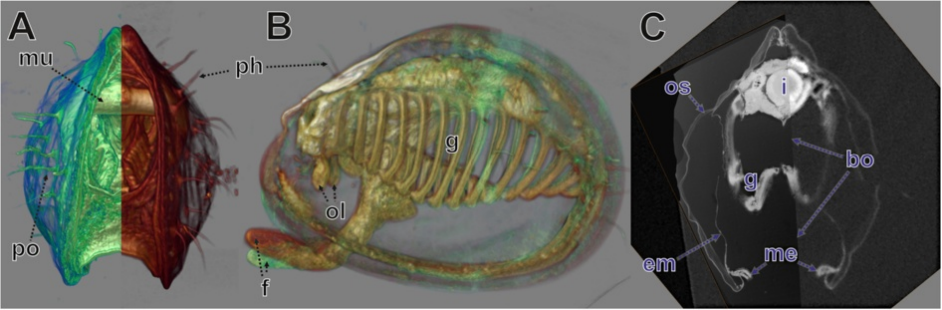
**Validation of co-registration by volume rendering and ortho slices.** LM serial section (registration adjusted) and microCT data (original registration retained) sets displayed by volume rendering **(A, B)** and slices **(C)** in AMIRA® **A**. View from posterior, left side (greenish) LM serial section data, right side (brownish) microCT data, both datasets visualized with the *volren* module and opposite side clipped. **B**. View from the left side. Datasets like in A but in full, transparency intensified by lowering *alphascale* values. Note deviation of foot tips. **C**. View from posterior, left side *orthoslice* of LM serial section data, right side *obliqueslice* of microCT data aligned with LM *orthoslice*. bo, border between orthoslices; em, external plus pallial cavity epithelium; f, foot; g, gill; i, intestine; me, mantle edge; mu, muscle; ol, oral lappets; os, organic shell layers; ph, periostracal hairs; po, periostracum and periostracal structures.

**Figure 7:**
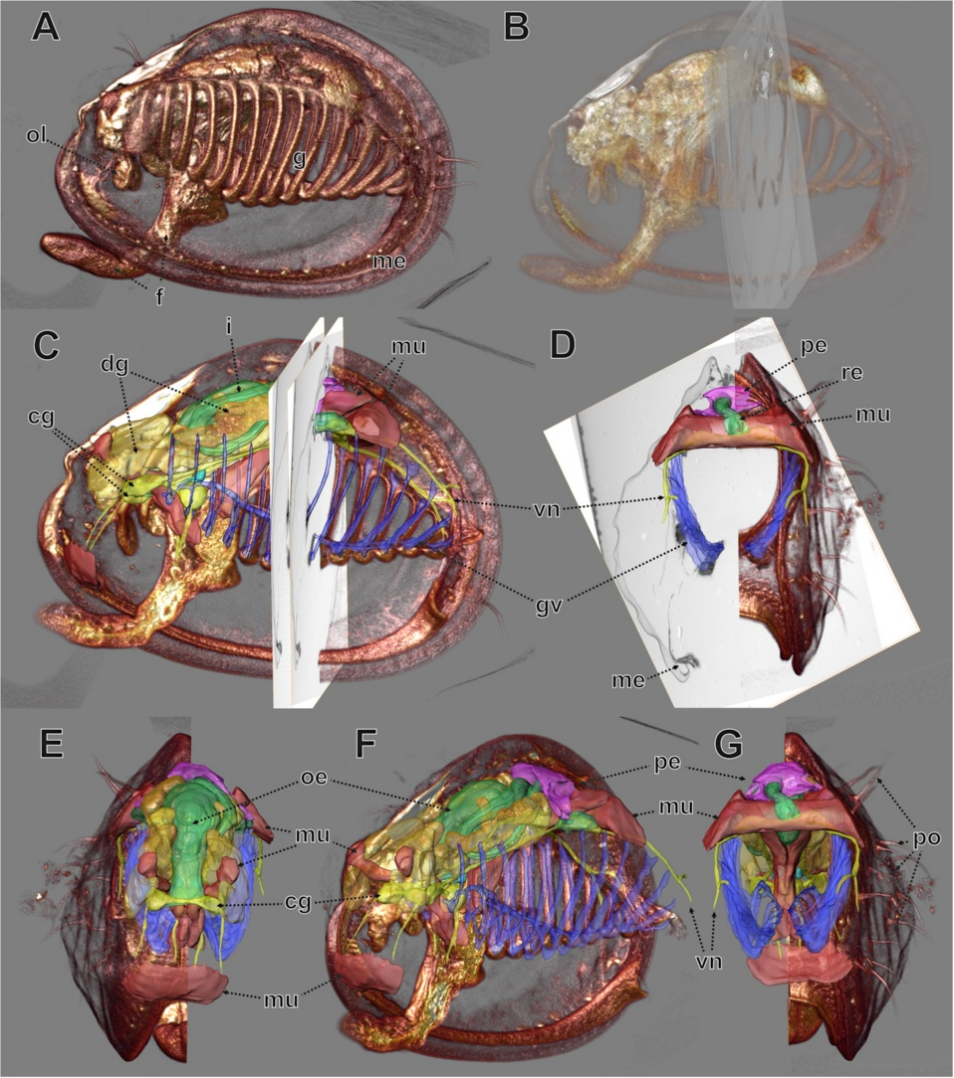
**MicroCT data (volume rendering) and LM data (surface rendering and *orthoslices*) combined.** AMIRA® software visualization. **A**. View from the left side, microCT data (*volren* module). **B**. MicroCT data with high transparency and transparent *orthoslices* of both LM sections used for TEM re-sectioning with TEM sections. **C**, **D**. Right half of microCT data, surfaces of various organs and *orthoslices* of LM sections used for TEM re-sectioning. **C**. View from left side. **D**. View from posterior. **F**–**G**. Same as **C**, **D** but without *orthoslices*. **E**. View from anterior. **F**. View from obliquely left. **G**. View from posterior. cg, cerebral ganglion; dg, digestive gland; f, foot; g, gill; gv, gill vessels; i, intestine; me, mantle edge; mu, muscle tissue; oe, oesphagus; ol, oral lappets; pe, pericardium; po, periostracum and periostracal structures; re, rectum; vn, visceral nerve.

**Figure 8:**
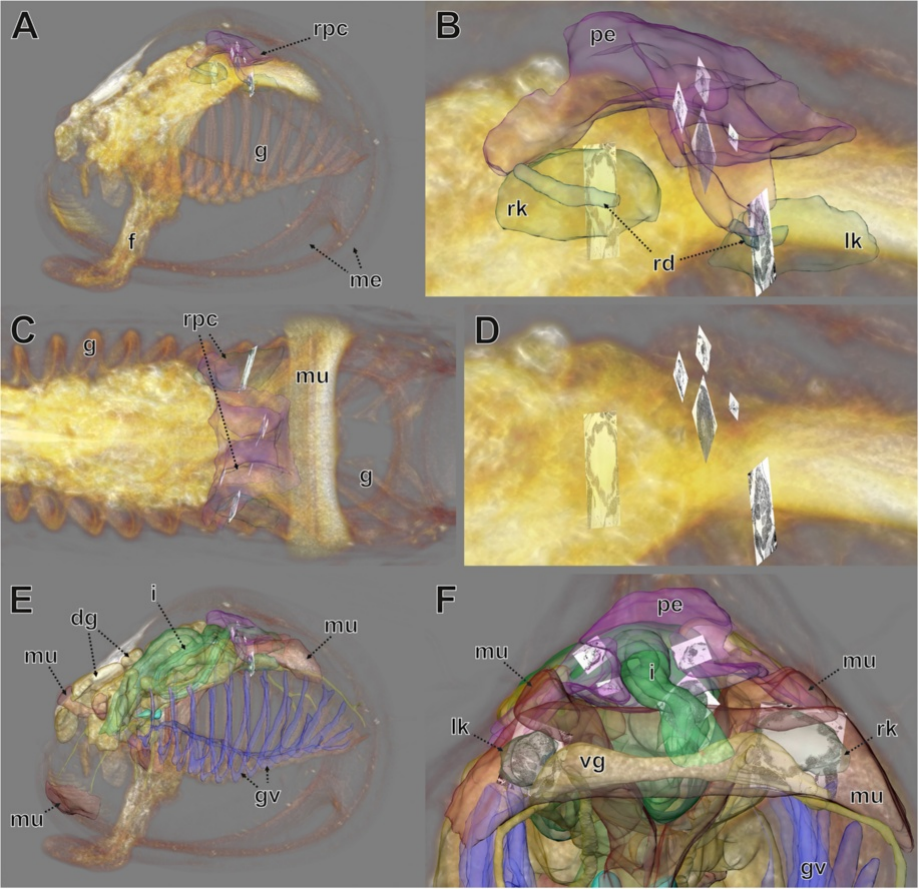
**MicroCT data (volume rendering), LM data (surface rendering), and TEM images (*orthoslices*) combined focusing on excretory system.***Volren* plus *voltex* modules used microCT data at high transparency (low alphascale values) in AMIRA®. **A**–**C**. MicroCT data, renopericardial complex and TEM sections **A**. View from the left side. **B**. Detail of **A**. **C**. View from dorsal (anterior is to the left). **D**. Same as B but without renopericardial complex. **E**, **F**. MicroCT data, various organs and TEM sections. **F**. Detailed view from posterior. dg, digestive gland;f, foot; g, gill; gv, gill vessels; i, intestine; lk, left kidney; me, mantle edge; mu, muscle tissue; pe, pericardium; rd, renopericardial duct; rk, right kidney; rpc, renopericardial complex; vg, visceral ganglion.

MicroCT, LM, and TEM data were displayed simultaneously in a single AMIRA® 3D scene (*Network*) (Figures 6, 7, 8) using a combination of different standard visualization devices for viewing volume data, polygonal surfaces, and individual slices. For volume data from microCT and LM sections both the *Voltex* (volume rendering via texture mapping) and the *Volren* module (volume rendering via ray casting) was used. Surface mesh files were rendered with the *SurfaceView* tool in *Direct Normals* mode. For visualizing LM and TEM sections *OrthoSlices* were used. In the case of TEM sections, the *OrthoSlice* was combined with a specifically adapted grayscale colormap with standard gray values and a transparency function where opacity for input gray values 0–9 is set to 0, and opacity in gray values 10–255 is set to 255. This yielded complete transparency in the surrounding area (black background, Figure 5C) and total visibility of the aligned TEM images.

## Results

### Technical – visualization

For the specimen treated in the present study, osmium tetroxide as routinely applied for TEM studies provides sufficient contrast for microCT investigation. By volume rendering with the Drishti software (Figure 2E–G) many structures of the specimen could be visualized adequately. This particularly concerns external structures, such as details of the shell periostracum (Figure 2E) and epithelial surface structures (e.g. the gills, Figure 2F). In addition some internal structures bearing specific density properties (e.g. shell resilium, shell muscles) could also be depicted. Even extremely delicate structures, such as the gill vessels (Figure 2G), could be rendered. Furthermore the embedding resin could by depicted, showing the trimming condition of the block at microCT scanning (Figure 2D).

All the different datasets (microCT, LM section series, TEM sections) could be aligned (co-registered) with the AMIRA® software (Figures 6, 7, 8). Both volumetric datasets (microCT and LM section stack) were neatly matched with regard to the main parts of the specimen after affine co-registration (Figure 6). Deviations were found to increase with distance to the specimen centre. These are most obvious at the periostracum hairs of the shell in the posterior area and anteriorly at the tip of the foot (Figure 6B).

In the viewer of the AMIRA® software all co-registered datasets can be viewed simultaneously. Here, a variety of settings allow changing or improvement of visual appearance of different components for better understanding and communication. These include adjustment of transparency (Figures 7, 8) of surface meshes, volumes or *OrthoSlices*, selective imaging of individual components (organs by surface meshes or section *OrthoSlices*) or clipping off parts of volumes or surface meshes (Figures 6, 7).

**Figure 9:**
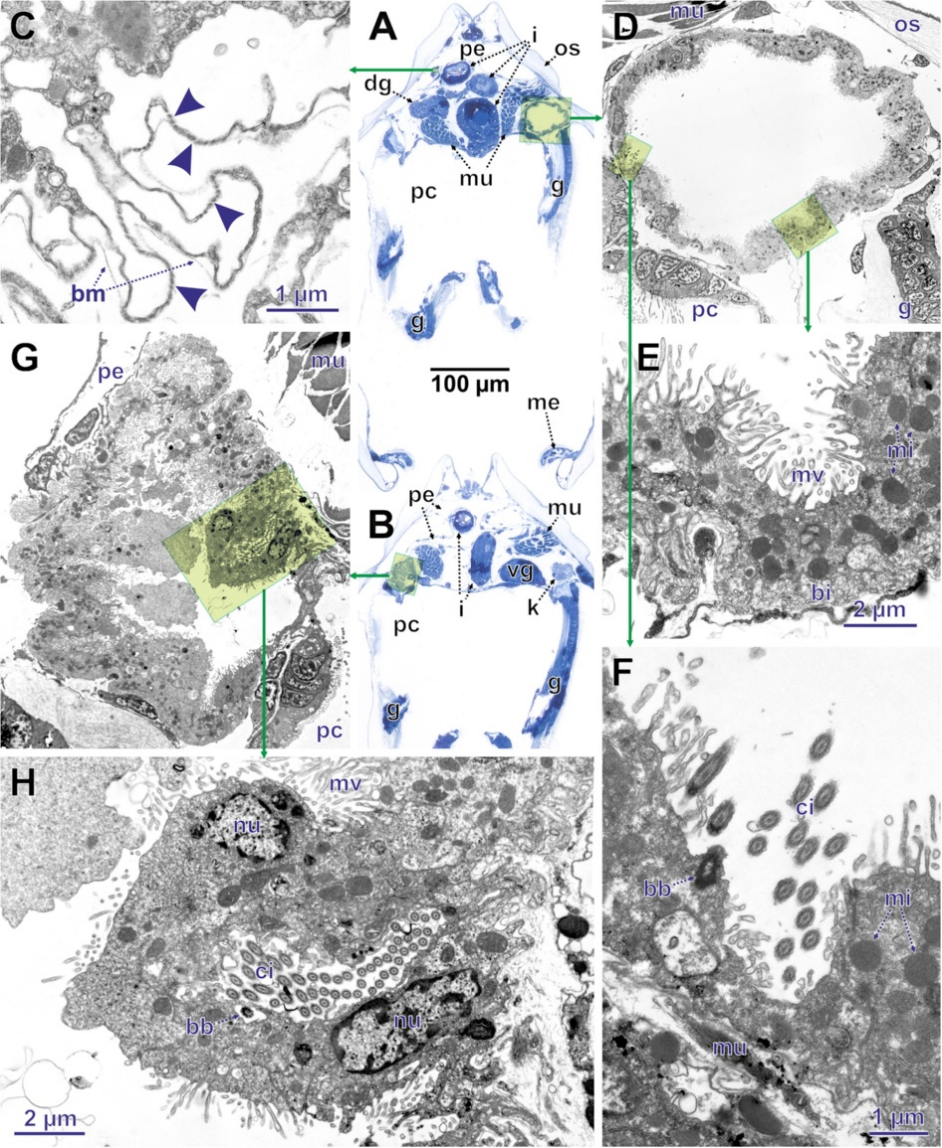
**LM and TEM re-sectioning results in conventional figure plate. A**, **B**. LM cross sections that were re-sectioned. **C**–**F**. TEM details of **A**. **G**, **H**. TEM details of **B**. Transparent yellowish rectangles with green edge show area enlarged in successive (green arrow) image with higher magnification. **C**. Detail of pericardial wall with ultrafiltration slits (arrow heads). **D**. Cross section through left kidney. **E**. Wall of kidney. **F**. Opening of renopericardial duct into kidney. **G**. Right kidney with most ventral end of renopericardial duct before opening into kidney. **H**. Most ventral end of renopericardial duct. bb, basal body; bi, basal infoldings; bm basal membrane; ci, cilia; dg, digestive gland; g, gill; i, intestine; k, kidney; me, mantle edge; mi, mitochondrium; mu, muscle tissue; mv, microvilli; nu, nucleus; os, organic shell layers; pc, pallial cavity; pe, pericardium; vg, visceral ganglion.

For all TEM images, the precise position within the corresponding LM images could be assessed (Figure 8). This enables preparation of conventional (2D) figure plates with interleaving these two image types (Figure 9). Thus fine structural details including their position within the organism can be provided simultaneously, which facilitates perception and presentation of structural relationships (Additional file 3).

### An example from morphology – the renopericardial complex

The renopericardial complex in the specimen investigated consists of a pericardium (Figures 7C–G, 8A–F, 9A,B) with anlagen of the internal heart components, and renopericardial ducts (Figures 8A,B, 9G,H) and kidneys (Figures 8A–F, 9A,D–H). The pericardium sits dorso-anteriorly adjacent to the posterior shell muscle in the posterior part of the animal (Figures 7C–G, 8A,E). It consists of a delicate endothelium (Figure 9G) that encloses a cavity. Laterally it exhibits conspicuous inward-directed pouches, which represent the anlagen of the internal heart components (atria, ventriculum). On both sides the pericardium bears latero-ventrally directed blind-ending extensions. Their most distal endings sit directly above the kidneys (Figure 8A–C).

The pericardial and kidney lumina are connected by the renopericardial ducts (Figure 8A,B, 9F-H). These are very delicate and descend from the median sides of lateral pericardial extensions. From the site of their emergence from the pericardial extensions the renopericardial ducts extend postero-ventrally, running medially adjacent to the kidneys (Figure 8B). The kidney walls are pressed inwards below the ducts (Figure 9G). They run ventrally until the ventral third of the kidneys, where they enter the kidneys. On the inside the renopericardial ducts are ciliated over their entire length until their opening into the kidneys (Figure 9F,H). The ducts are directed towards the kidneys with some of them protruding into the kidney lumina (Figure 9F).

The kidneys represent compact organs with relatively thick walls. In cross sections they are circular and they are somewhat elongated in the antero-posterior direction. On the ventral side they open via a porus into the mantle cavity.

The sites of ultrafiltration are large and located at the pericardial walls next to the inward-directed pouches (anlagen of heart components) of the pericardium (Figure 9A,C). These ultrafiltration sites are composed of slit-shaped openings separated by pedicles (Figure 9A). In overall shape the sites are folded and their basal side (external surface) is coated with a distinct basal membrane (extracellular matrix).

The cells of the kidney walls exhibit electron-dense cytoplasm with numerous mitochondria. Basally these cells bear a very dense infolding system (Figure 9E). The apical side (towards the lumen) has numerous partly branched microvilli (Figure 9D–H).

## Discussion

### Merits of the workflow

The correlative examination of a small biological specimen offers a number of advantages in the analysis, visualization, and validation of results. The option of topographical correlation of high resolution areas to the morphology of the whole specimen provides significant benefits. In many TEM investigations only isolated sections instead of complete series are examined in order to avoid excessive effort. This may lead to serious misinterpretations, because of missing information on the surrounding tissue. Furthermore, problems frequently occur during TEM sectioning resulting from, for example, inhomogeneous polymerization of the resin or non-cuttable contents, such as sand grains. These may lead to the loss of portions of the sample. In such cases the CT data are a highly valuable backup containing the information for recovering the overall organization of the specimen. As demonstrated by the excretory organs of our specimen, fine structural details of isolated TEM areas can be combined with other datasets to achieve an overview of the organ complex without missing significant information. Correlative morphology is also of great advantage for effectively communicating results with the help of 3D visualization. In our example the display of (TEM) sections in the form of *OrthoSlices* within a combination of volume and surface renderings of other datasets are highly efficient for explaining the position of minute structures within the entire organism. Finally, datasets may be important for validating other datasets. For example, aligning images of LM sections is usually performed on a section-to-section basis. Since information used for alignment is only from adjacent sections, the overall shape may become inaccurate with distortions or dislocations propagated and amplified over the length of the specimen. Since microCT datasets do not contain inaccuracies of that kind, they can be utilized for validation and improvement of serial section alignment. In general, microCT information is extremely valuable for all types of specimens to be sectioned. This also includes larger specimens, which are routinely embedded in e.g. paraffin or methyl methacrylate. Since such samples are too large for osmium tetroxide postfixation, they have to be stained using an alternate contrast agent such as elemental iodine or phosphotungstic acid [17]. In these cases knowledge of internal morphology can be used for identifying regions of interests (ROI), to which the specimen can be trimmed prior to sectioning.

Among the most important merits of our combined approach is the reduction in labor and general effort required for fine structural analysis. At present there is a considerable range of methods for structural (3D) examination at the (sub-)cellular level. These include electron tomography, serial block face scanning electron microscopy (SBF-SEM) (3View), focused ion bean scanning electron microscopy (FIB-SEM), and serial section TEM (see e.g. [28] for a review). However, all these techniques are very costly in labor or equipment. Examination of a specimen of the size used in the present study purely by TEM examination would take many weeks of painstaking sectioning and many longTEM sessions. SBF-SE und FIB-SEM are very expensive to operate and the volumes that can be examined by FIB-SEM and electron tomography are very small (see e.g. [28]). Thus no entire organ system of our example specimen could be assessed by these methods. Although our approach involves a variety of different equipment and procedures, we maintain that it is actually quite economical with regard to both manpower and cost.

### Merits of the analysis of the present example – the juvenile *Mytilus galloprovincialis*

Based on co-registered datasatets, we could analyze the fine structure of the major components of the renopericardial system and the general morphology of the renopericardial and other organ systems and combine this with a data set showing the entire organism of a juvenile *Mytilus galloprovincialis*. The level of detail/resolution corresponds to the method of analysis (Additional file 4). The fine structural data was gained by subsequent TEM examination of LM sections; the general morphology of the renopericardial and other organ systems was obtained by segmentation of the LM section series with surface rendering. The arrangement of the different organ systems in respect to the entire specimen was visualized by microCT data. Accordingly, the spatial relations among the major components of the specimen are clearly recognizable.

Although this is not the primary purpose of the present study, the results on the renopericardial system can be discussed by comparing them to previous studies on *Mytilus* and data on other bivalve molluscs: the fine structural details regarding the kidney epithelium or pericardial ultrafiltration sites correspond well to previous data for adult *Mytilus* (e.g. [29,30]) and also to data on other molluscs (e.g. [31,32]). In contrast, there is a major difference in our results concerning the renopericardial duct: while previous investigations [29,33]) show a broad connection entering the kidney dorsally, we found that this duct is delicate, runs adjacent to the kidney wall and opens into the kidney at a medioventral position. At present it is unclear whether this difference is due to the different age of the specimen (0.8 mm juvenile in the present study vs a several cm adult in previous studies) or to faulty observations of White [33], which also served as basis for the description of Pirie & George [29]. It appears possible that they mistook a lateral branch of the pericardium reaching towards the kidney – a structure that is present in our specimen – as renopericardial duct and overlooked the actual duct which was too delicate to be resolved by dissection. This should to be clarified by a further study on adult *Mytilus* specimens.

### TEM-processed samples and microCT

X-ray absorption of animal soft tissues is very poor. Accordingly, if the specimen is not stained with a contrast-enhancing substance it can hardly be visualized in conventional computer tomography, though it could be imaged with phase contrast synchrotron microCT [34,35]. Initial attempts for contrasting soft tissues for conventional microCT concentrated on heavy metals stains such as osmium tetroxide (e.g. [16,36,37]). More recently, other less toxic substances like iodine-based contrast media, were found to provide very good contrast as well ([12,17,38]) equal to that of osmium tetroxide [39]. For our approach it is highly convenient that osmium tetroxide is routinely applied for postfixation of material for TEM: this postfixation provides reasonable contrast for microCT while the X-ray absorbance of epoxy resins used as embedding media in electron microscopy is generally low [17,18,40]. Thus microCT scanning could be considered as a routine procedure before TEM investigation, because it provides valuable additional information and compared to the TEM procedures it causes little extra time effort at tolerable operating costs (during the last years microCT scanners became much more frequent in labs and thus broadly available to researchers). This is also true for methods such as SBF-SEM (3View) [41] and FIB-SEM [42], where samples are stained en bloc prior to embedding in resin. This differs slightly from conventional TEM procedures prior to embedding in that the osmium tetroxide treatment is usually intensified and additional metal compounds are applied to enhance contrast. This treatment very likely also increases X-ray absorption. Consequently, such specimens appear particularly suitable for initial X-ray tomographic examination.

### TEM re-sectioning of resin LM sections

Re-sectioning of LM sections has been attempted since the early days of TEM [43], as it permits precisely tracking down the position of the structures to be examined and thus reduces the amount of work. There are numerous protocols for re-sectioning (e.g. [20,26,44-46] and references therein) available. These differ with regard to (a) the detaching of the LM section from the slide and (b) the way of attaching the section to the new empty block. (a) Detachment of the section was performed by either polymerizing the new block directly to the section, which was still attached to the slide (e.g. [20,43,47]), or in some cases by flash-freezing the slide (e.g. [20,45]). (b) Bonding the section to the new block was reinforced by applying adhesives like epoxy resins [43,48] or specific glues [20,46,49]. In the present study we applied the simplest procedures for detaching sections and re-attaching them by simply drying the section. This is essentially the procedure suggested by Campbell & Hermans [26]. The only difference concerns lifting and transferring of the sections. We carried this out even more simply than Campbell & Hermans [26], with the tip of a preparation needle (which fulfils this function perfectly) instead of a wire loop or TEM grid. The use of adhesives for bonding LM sections to the new block did not seem advantageous to us, since sections might not become tightly adjacent to the surface of the empty block. By applying adhesives the previously sectioned plane could possibly become lost, which might not be a problem for relative thick sections (e.g. [20]: 10 μm). However, for 1.5 μm sections as used in the present study, ultrathin section output would probably worsen. We suspect the use of adhesives would lead to fewer usable TEM sections per LM section or that only parts of LMs would get sectioned.

Re-sectioning of LM sections for TEM has only rarely been applied for zoological-morphological purposes, which seems remarkable since numerous protocols suggest this. Nevertheless, it should be particularly useful for small invertebrates since individual structures and regions of interest can be selected precisely, and the effort in both sectioning and TEM examination can be kept very low. Another frequently applied method of combining LM with TEM in the same specimen is alternating LM and TEM sectioning. Our own experience [23,24] has shown that LM re-sectioning is much more efficient, because alternating sectioning requires repeated changes of the equipment (e.g. knife, microtome) during sectioning and the number of sections to be examined with the TEM is significantly higher. It should be mentioned that our re-sectioning strategy has one significant limitation: 3D reconstruction at the fine structural level (such as performed by e.g. Cardona et al. [50]) is nearly impossible. This is because the maximum z-depth of individual TEM section stacks is limited to the thickness of LM section (1.5 μm for the present example) and most structures of interest are larger than that. Another shortcoming is that the LM section series has to be left uncovered until selected sections become removed for TEM re-sectioning. However, this problem should not be overestimated: overall images of sections are of reasonable quality (Figure 9A,B) and details are analyzed by TEM examination anyway.

### AMIRA®s affine registration module

The *Affine registration* module of AMIRA® accomplishes stack-to-stack registration only. Hence, it cannot compensate for misalignments within the LM stack originating from the previous serial section alignment. Since our results (comparison of the LM stack with the reference microCT stack) experience mainly peripheral misalignments (Figure 6B), this method of registration is sufficient for our example. If misalignments were to exceed a tolerable amount, it would be required to step back in the procedure and repeat or refine the serial section alignment in affected regions of the image stack. The *Affine registration* module offers a range of different *metric* options to perform registration (for information on multimodal image registration see e.g. [51,52]). For the presented specimen all available metrics worked equally well. Transformation may also involve isotropic or anisotropic scaling; anisotropic scaling might be especially beneficial for the alignment of LM sections, because it could balance an inaccurate section thickness provided by the microtome (e.g. 0.95 μm instead of 1 μm) by re-scaling only the z-dimension of the image stack. In the presented example, section thickness appeared to be accurate, so anisotropic scaling was not required.

The quality of image registration depends very much on the properties of the image data. The *Affine registration* tool was designed to handle data from different imaging sources such as CT or MR. Nevertheless, we found that the degree of similarity between datasets has a major effect on registration results. MicroCT images show bright objects on dark background, while LM sections show dark objects on bright background. To maximize similarity we (1) inverted the LM section stack and (2) used a Gaussian blurring filter to decrease the level of detail of LM images [3]. Applying these adjustments, we achieved the best results.

### Identical objects or sub-regions for co-registration

The current workflow is designed for datasets that both show exactly the same specimen (reference stack and stack to be registered), as was the case in the presented specimen for which the microCT and LM dataset contained the same sample with identical boundaries. In contrast, there can be datasets that cover different amounts of a specimen. For example, there might be a microCT scan of an entire specimen, and based on the information of microCT data the block is trimmed to a specific ROI before sectioning. Thus the LM data volume represents a sub-region of the original microCT-scanned object, and automatic registration with the *Affine registration* might fail even after thorough manual pre-alignment. In that case, cropping the reference volume to a similar region as the physically restricted LM dataset might solve problems for co-registration.

### Rigid or elastic registration

In the presented workflow, both the 3D stack-to-stack registration (microCT and LM) and the 2D section-to-section registration (EM and LM) is done by using rigid registration and scaling, leading to adequate results showing little impairment from geometric distortions. However, in principle it would be possible to include elastic registration algorithms for both procedures. Epoxy resin sections at LM thickness usually show very little geometric distortion in terms of stretching or shrinkage, which allows rigid registration for the stack-to-stack registration. Geometric deformations are a more serious problem for TEM sections where elastic registration for volume generation is required (e.g. [53,54]). This type of elastic registration could be also beneficial for the refinement of TEM to LM sections.

## Conclusions

The procedure of co-registration of datasets from different imaging modalities offers new opportunities for understanding and communicating structural relationships within organisms and tissues. Thus the correlative use of different microscopic imaging techniques will likely become more widespread in morphological and structural research in zoology. Classical TEM serial section investigations are extremely time consuming and hence cannot keep up with modern 3D methods such as CLSM where results are achieved much faster. Re-sectioning of LM sections seems suitable for speeding up TEM-based examination substantially. At the same time, microCT stands to become a key method for complementing ultrastructural examinations. Scanning specimens is relatively straightforward and lab-based (as opposed to synchrotron) X-ray microscopy systems are now available to most research institutions. It seems promising to apply microCT in addition to block face scanning methods (SBF-SEM, FIB-SEM) for the option to view the limited area captures by these methods in wider context.

## Competing interests

The authors declare that they have no competing interests.

## Authors’ contributions

SH performed AMIRA procedures including registration of datasets and 3D scenery visualization, and drafted parts of the manuscript. NB carried out practical laboratory procedures including sectioning and TEM procedures. TS performed LM image acquisition and image segmentation for polygon models. BR initialized and coordinated this study, performed 3D rendering in Dristhi, and drafted parts of the manuscript. All authors contributed to the content and writing of this paper and approved the final version of the manuscript.

## Supplementary Material

Additional file 1 **Specimen embedded in block, visualized via volume rendering with DRISHTI.** The microCT dataset was visualized by volume rendering in DRISHTI applying transfer functions in the *2D histogram*. Individual color and transparency settings for multiple transfer functions permitted discerning tissues with different density attributes. Cropping of the specimen was done using *ClipPlanes*. Click here for file

Additional file 2 **Automatic affine registration in AMIRA®.** Fine co-registration performed automatically by the *AffineRegistration* module, followed by a rotation of the object to show the quality of alignment. Click here for file

Additional file 3 **MicroCT data, LM data, and TEM images combined in an AMIRA® animation sequence.** In the viewer of the AMIRA® software all co-registered datasets can be viewed simultaneously. Different settings allow changing or improvement of visual appearance of different components for better understanding. Click here for file

Additional file 4 **Different levels of resolution achieved by combination of microCT, LM, and TEM.** This AMIRA® zoom-in animation shows the different levels of resolution achieved by our correlative approach. Click here for file
